# A Plan for the Study of Man

**Published:** 1907-09

**Authors:** Arthur MacDonald

**Affiliations:** Washington, D. C., Honorary President of the “3rd International Congress of Criminal Anthropolgy,” of Europe


### A PLAN FOR THE STUDY OF MAN.

By Arthur MacDonald, Washington, D. C.,
Honorary President of the "3rd International Congress of Criminal Anthropolgy," of Europe.

The greatest of all studies is that of man himself as he is today. A scientific investigation of man must be based primarily upon the individual, who is the unit of the social organism.

If we are ever to have sufficient definite knowledge of living human beings that may become a science, it can only be done by the careful study of large numbers of individuals. The more thorough the study and the larger the number, the more useful such investigation can be made to society.

As in machinery we must first repair the little wheels out of gear, so in society we must first study the criminal, crank, insane, inebriate or pauper who can seriously injure both individual and community. Thus a worthless crank, killing a prominent citizen, can paralyze the community. The injury from such action is often beyond calculation. Governments pay out millions to catch, try, and care for criminals, but gives very little to study the causes that lead to crime.

The study of man, to be of most utility, must be directed first to the causes of crime, pauperism, alcoholism and other forms of abnormality. To do this the individuals themselves must be studied. As the seeds of evil are usually sown in childhood and youth, it is here that all investigation should commence, for there is little hope of making the world better if we do not seek the causes of social evils at their beginnings.

The most rigid and best method of study of both children and adults is that of the laboratory, with instruments of precision in connection with sociological data. Such inquiry consists in gathering sociological, pathological, and abnormal data as found in children, in criminal, pauper, and defective classes, and in hospitals, schools and other nstitutions. Such experiments or measurements should be made as are of interest not only to sociologists, psycho-physicists and anthropologists, but also to physiologists and pathologists.

But, it may be asked, what as to the utility of studying such questions? We think it is not only useful, but there is great need of such investigation. We should like to inquire, for instance, as to the utility of studing rocks and plants, arranging them, making chemical analyses of them, etc., if it is not to give a deeper knowledge of them and thereby learn more about our planet? So the patient and extended study of man, especially children, is to gain more definite knowledge about him and a deeper insight into his nature. The time has certainly come when man, as he is, should be studied as much as nature.

Much money has been given and great interest manifested for the discovery of new chemical elements or the search for unknown planets. We erect statues and found art galleries at great expense. These things may not all be immediately useful. Indeed, the highest art spurns even the idea utility; and yet when it is proposed to study a child thoroughly to gain an insight into its nature, to find the causes of its defects, so that we may protect it and help it to become a good citizen, the utilitarian cry is heard. The time has come when it is important to study a child with as much exactness as we investigate the chemical elements of a stone or measure the mountains on the moon.

### SOME CONCLUSIONS AS TO CRIMINAL MAN.

The following statements as to the criminal are not based upon experimental research so much as upon the experience of those who have studied criminals directly or who have had practical control of large numbers in prisions or reformatories:

1. The prison should be a reformatory and the reformatory a school. The principal object of both should be to teach good mental, moral, and physical habits. Both should be distinctly educational.

2. It is detrimental financially, as well as socially and morally, to release prisoners when there is a probability of their returning to crime; for in this case the convict is much less expensive than the ex-convict.

3. The determinate sentence permits many prisoners to be released who are morally certain to return to crime. The indeterminate sentence is the best method of affording the prisoner an opportunity to reform without exposing society to unnecessary dangers.

4. The ground for the imprisonment of the criminal is first of all because he is dangerous to society. This principle avoids the uncertainty that may rest upon the decision as to the degree of freedom of will; for upon this last principle some of the most brutal crimes would receive a light punishment. If a tiger is in the street, the main question is not the degree of his fredeom of will or guilt. Every man who is dangerous to property or life, whether insane, criminal, or feeble-minded, should be confined, but not necessarily punished.

5. The publication in the newspapers of criminal details and photographs is a positive evil to society, on account of the law of imitation; and, in addition, it makes the criminal proud of his record, and develops the morbid curiosity of the people; and it is especially the mentally and morally weak who are affected.

6. It is admitted by some of the most intelligent criminals, and by prison officers in general, that the criminal is a fool; for he is opposing himself to the best, the largest, and the strongest portion of society, and is almost sure to fail.

### *REASONS WHY FEDERAL, STATE, AND CITY GOVERNMENTS AND ALSO PRIVATE ENDOWMENT SHOULD ESTABLISH LABORATORIES FOR THE STUDY OF THE ABNORMAL CLASSES,*

being in part a Resume of Hearings given by the writer, before the Finance Committee of New York State Senate and the Judiciary Committee of the United States House of Representatives.

BILL

To establish a laboratory for the study of the criminal, pauper, and defective classes.

Be it enacted by the——, That there shall be established in the —— a laboratry for the study of the abnormal classes, and the work shall include not only laboratory investigations, but also the collection of sociological and pathological data, especially such as may be found in institutions for the criminal, pauper, and defective classes, and generally in hospitals and other institutions. Said laboratory and work shall be in charge of a director, who shall be appointed by the ——, and shall receive a salary of three thousand dollars per annum. He shall make a report once a year, directed to the ——which, with the approval of that officer, shall be published. For the aid of the director there shall be one psychologist, at two thousand dollars; one translator, at one thousand two hundred dollars; one stenographer and typewriter, at one thousand dollars, and one mechanic, at nine hundred dollars. For the proper equipment of and carrying on the work of said laboratory and the rental, if necessary, of suitable rooms therefor, there is hereby appropriated, out of any money in the Treasury not otherwise appropriated, the sum of five thousand dollars, or so much thereof as may be required.

It is not expected that such an extensive field of work, as indicated in bill, be undertaken at the outset. It is therefore suggested that a beginning be made with the crimnal classes. If necessary, in order to pass bill, it might be reduced, the minimum being a director at one thousand dollars and two hundred dollars for laboratory. Even with this very small total appropriation of twelve hundred dollars, a beginning can be made.

An idea pervading the bill is that Cities, States, and Nations should look after the moral health of the people with as much scientific foresight as they do the physical health of the people. Such work is fundamentally humanitarian. The task is large enough to require the aid of all forms of government and also of private endowment, and it is due time that such efforts be made; for the official statistics of the leading countries of the world show, that within the last thirty or more years, crime, suicide, insanity and other forms of abnormality have been increasing relatively faster than the population.

PRACTICAL RESULTS.

In every new line of work it is impossible to know in advance the practical results, but it is an axiom of science and sociology that no evil can be permanently lessened unless its causes be studied first and that such study poduces practical results. Science has demonstrated this fact again and again.

If the student seeking the cause of cholera had been required to state in advance whether he could lessen or cure cholera or not, after he had found its cause, and had been refused aid because such an uncertain work was deemed impracticable, cholera might have been continuing its ravages up to the present time.

Although no cure has been found, yet the knowledge gained from the study of the cause of this disease has enabled science to prevent it to such an extent that it is now feared no more. To insist on this practical-result requirement in the study of social disease called crime is as unreasonable as it would have been in the case of cholera, and more so, for the ravages of crime exceed many times those of any physical disease.

If the practicability of a new plan of work be a matter of opinion, that opinion has most weight, which comes from those dealing first hand with some phase of the work. Such opinion is indicated on the last two pages of this article, under the head of "Summary of Indorsements of Work."

The main purpose of this bill is to study the causes of crime, pauperism, alcoholism, defectiveness, degeneracy and other forms of abnormality, with a view to lessening or preventing them. It is assumed that every citizen is interested in the purpose of such a bill.

In addition to this general scope of the bill there are some other direct ends which eventually the bill is expected to accomplish:

1. To gain more trustworthy knowledge of social evils. Such knowledge would furnish a basis for modifying defective laws, adapting them to present conditions.

2. To find whether or not there are any physical or mental characteristics that distinguish criminal children from other children. Such knowledge would make it possible to protect children in advance and lessen the chances of cantamination.

3. To find whether or not there are any physical and mental characteristics that distinguish habitual from occasional criminals. Such knowledge would enable the community to protect itself in advance from habitual criminals and assist prison officials in preventing them from contaminating other criminals.

4. Exhaustive study of single typical criminals, which represent a large number, will give definite knowledge as to just how men before criminals and to what extent their surroundings influence them as compared with their inward natures. This would make possible a rational application of remedies for these evils.

5. More exact knowledge of the abnormal classes will enable us to manage them better in institutions. Such studies will bring men of better education and training in control of the institutions, and increase interest in the professional study of these classes.

6. Proper and full statistics of the abnormal classes will alone justify this work. Merely skeleton statistics on this subject are sometimes gathered by governments.

7. As most of the inmates of reformatories and prisons are normal, any knowledge gained about them will be useful to the community at large. A scientific study of moral character can, for instance, be conducted best in such institutions.

8. To summarize and combine results already gathered by City, State and Federal institutions and governments, encouraging uniformity of method in collecting data and making such data useful generally.

9. To lessen the enormous expense to governments of the abnormal classes by study of the causes of the evils that involve such expense.

10. To appoint moral health officers (as well as medical) to study causes and provide measures for protecting City, State and Nation from crime, pauperism, alcoholism, degeneracy, defectiveness and other forms of abnormality.

Since the care, support, and direction of inmates of institutions for the abnormal and weakling classes are under City, State and Federal contol, the scientific and sociologic study of these inmates naturally falls under the same control.

The great progress already made by governmental scientific investigation of physical disease suggests governmental application of similar methods in the study of moral and social disease, the necessity of preventing or lessening which is much more urgent.

One reason why so many professional organizations dealing first hand with some phase of this work support this measure is that they think it is time that governments begin a serious study of those social evils which are their greatest enemies. Many worthy efforts are being made to lessen social evils, but they are mostly palliative, and do not go to the root of the matter.

One feature of this work, of interest to all lovers of truth, is the application of the results and methods of anthropology, psychology, medicine, sociology, and other sciences to the abnormal and weakling classes, thus constituting a new synthetic study which may bring out truths that apply as well to normal man as to abnormal man; for in the case of penal institutions most of the inmates, as already stated, are normal, their crime being due to unfortunate surroundings and not to their inward natures. Even really abnormal persons, that is, those positively abnormal in at least a few respects, are nevertheless normal in most things, so that whatever be found true of them is to a large extent true of all persons. Though such results be incidental, they may be none the less important. 1

As an illustration of the application of pscho-physics to sociology, a minor study of sensibility to pain in persons of different social and mental conditions, is here added.

### MEASUREMENTS OF PAIN.

We give some of our results of pain measurements on different classes of individuals, in all, 2311:

1. In general the sensibility to pain decreases as age increases. The left temple is more sensitive than the right. This accords with former experiments that the left hand is more sensitive to pain than the right hand.

2. Girls in private schools, who are generally of wealthy parents, are much more sensitive to pain than girls in the public schools. It would appear that refinements and luxuries tend to increase sensitiveness to pain. The hardihood which the great majority must experience seem advantageous. This also accords with our previous measurements, that the non-laboring (professional and mercantile) classes are more sensitive to pain than the laboring classes.

3. University women are more sensitive than washerwomen, but less sentitive than business women. There seems, however, to be no necessary relation between intellectual development and pain sensitiveness. Obtuseness to pain appears to be due more to hardihood in early life.

4. Self-educated women, who are not trained in universities,

---

1. For further points as to scope of work, see "Man and Abnormal Man," (by the writer) including a study of children, in connection with bills to establish laboratories under State and Federal governments in the study of the criminal, pauper and defective classes, with bibliographies. Senate Document No. 187, 58th Congress 3d Session. 780 pages, 8°. Washington, D. C.

This document might be obtained gratis on application to any United States Senator or Representative.

are more sensitive than business women. The greater sensitiveness of self-educated women as compared with university women may be due to the overtaxing of the nervous system of the former in their unequal struggle after knowledge.

5. Girls in the public schools are more sensitive at all ages than boys. This agress with the results of our previous measurements that women are more sensitive to pain than men. But this does not necessarily refer to endurance of pain.

These measurements of least disagreebleness, or of threshold of pain, are approximate measurements of the combination of nerve, feeling, and idea. Which one of these elements influences the combined result most would be difficult to say.

Below is a description of the temporal algometer (designed by the writer) used in the experiments:

TEMPORAL ALGOMETER.

It consists of a brass cylinder B F, with a steel rod C running through one of the ends of the cylinder. This rod is attached to a spring, with a marker E on the scale A; this scale is graded from 0 to 4,000 grammes. The brass disc. D is 15 millimeters in diameter; a piece of flannel is glued to its surface, so as to exclude the feeling of the metal when pressed against the skin, thus giving a pure pressure sensation. The whole instrument is 30 centimeters in length.

In using this algometer it is held in the right hand at B, by the experimenter, who stands back of the subject and presses the disc D against the right temporal muscle, and then he moves in front of the subject, where he can conveniently press the disc against the left temporal muscle. These muscles are preferred because no trade or profession materially affects them. They are also conveniently situated.

As soon as the subject feels the pressure to be in the least disagreeable the amount of pressure is read by observing the marker E on the scale A. The subject sometimes hesitates to say just when the pressure becomes in the least disagreeble, but this is part of the experiment. The purpose is to approximate as near as possible to the threshold of pain.

The object here is not to see how much pain can be endured, as some have supposed, but the very opposite—that is, when the pressure becomes the least bit disagreeable the subject is to say so at once. The pressure is increased very gradually, so that the subject can decide more exactly when the first unpleasant pressure sensation arises. In all the experiments made, no one ever complained of being hurt in the least.

Instead of the temporal algometer being an instrument to make pain, as some have imagined, it may assist in telling us more about the nature and causes of pain, and thereby furnish a way of prevention or amelioration of pain.

### *SUMMARY OF INDORSEMENTS OF WORK.*

The general purpose of the work is to establish laboratories under City, Federal and State control, and also under private endowment, for the study of the criminal, pauper, defective and other abnormal classes, with a view to lessening these social evils by investigations of their causes.

These indorsements are not merely formal, but committees were appointed to examine the work and report to their associations resolutions, with the result that the work has received scientific, medical, legal and religious support of highest rank. This will be seen from the following:

INDORSEMENTS OF SCIENTIFIC AND MEDICAL ASSOCIATIONS.

"Ve Congres International d' Anthropolie Criminelle," consisting of leading University specialists in Europe.

The Pedagogical Society of the University of Moscow.

The Anthropological Society of Bombay, India.

The Medico-Legal Society of New York.

Six National Medical Societies:

The American Medical Association.
The Association of American Medical Editors.
American Medico-Psychological Association.
The Association for the Study and Cure of Inerbriety.
The American Larnygological Society.
The American Electro-Therapeutic Society.

Twenty-five State Medical Societies: Connecticut, Colorado, Idaho, Indiana, Kansas, Kentucky, Louisiana, Minnesota, Medical Society of the Missouri Valley. Mississippi Valley Medical Association, New England Pschological Society of Alienists, New England Hospital Society, Medical Association of Central New York, North Dakota, New Jersey, Pennsylvania, Sea-Board Medical Association, Texas, Tri-state Medical Society of Alabama, Georgia, and Tennessee, Utah, Virginia, West Virginia, Wisconsin, and District of Columbia.

### LEGAL ASSOCIATIONS INDORSING WORK.

The American Bar Association, the most representative body of the legal profession in the United States.

Four State Bar (Indiana, Kansas, Louisiana, New Mexico) and three City Bar Associations (Indianapolis, Lancaster, Murfreesboro).

### RELIGIOUS AND OTHER ASSOCIATIONS INDORSING WORK.

Twenty-five Presbyteries in California, Colorado, Illinois, Iowa, New York, Ohio, Pennsylvania, Washington, and Washington City, D. C.

Three State (Massachusetts; Michigan, and New York) and one District Universalist Conventions.

One State (Minnesota) and three District (Massachusetts) Unitarian Associations.

One Reform Church Classis, three Baptist and other Religious and Charitable Associations.

Two State Conferences of Congregational Churches (Rhode Island and Maine) and three State Dioceses (Michigan, Central Pennsylvania, and North Carolina).

### INDORSEMENTS OF AMERICAN AND EUROPEAN SPECIALISTS.

Fifty-five American and twenty European specialists have written personal letters indorsing work. Most of these specialists are University professors. The others are engaged on the practical side of the work.

127 A Street, N. E., Washington, D. C.

